# Considering vocational training as selection criterion for medical students: evidence for predictive validity

**DOI:** 10.1007/s10459-022-10120-y

**Published:** 2022-07-06

**Authors:** Dorothee Amelung, Simon Zegota, Lia Espe, Tim Wittenberg, Tobias Raupach, Martina Kadmon

**Affiliations:** 1 Medical Faculty, Heidelberg University, Im Neuenheimer Feld 110, 69120 Heidelberg, Germany; 2 Medical Faculty, Göttingen University, Göttingen, Germany; 3 Medical Faculty, Bonn University, Bonn, Germany; 4 Medical Faculty, Augsburg University, Augsburg, Germany

**Keywords:** Admission criteria, Medical student selection, Medical school, Vocational training, Professional experience, Multicenter study

## Abstract

Prior work experience in a relevant medical profession is an important admission criterion currently used at many German medical schools in addition to cognitive criteria. In other countries, work experience is often considered in later admission stages (e.g., interviews with pre-selected subgroups of applicants). However, evidence for its predictive validity for study success in addition to cognitive admission criteria is currently lacking. We therefore assessed whether completed vocational training in a relevant medical profession can predict study performance in the first two years of study in addition to cognitive admission criteria. Admission and study performance data of all currently enrolled medical students at two German medical schools (Göttingen and Heidelberg) beginning with the 2013/14 cohort were retrospectively analyzed. Cognitive admission criteria in our sample were GPA grades and a cognitive test (“Test für Medizinische Studiengänge”, TMS). We defined the study outcome parameter as the mean percentile rank over all performance data points over the first two years of study for each location, respectively. A multi-level model with varying intercepts by location, GPA, TMS, vocational training, and sex as predictors accounted for 14.5% of the variance in study outcome. A positive predictive association with study outcome was found for vocational training (ß = 0.33, p = .008) beyond GPA (ß = 0.38, p < .001) and TMS (ß = 0.26, p < .001). Our results support the use of prior vocational training as a selection criterion for medical studies potentially adding predictive validity to cognitive criteria.

## Introduction

German medical school applicants can increase their likelihood of being accepted at many medical schools in Germany if they successfully complete relevant vocational training in a medical field (e.g. as an emergency paramedic or nurse) prior to application. Almost all German medical schools currently consider this criterion when preparing their ranking lists, in addition to their average score obtained in the German “Abitur” (roughly equivalent to Grade Point Average (GPA) scores in the USA) and other criteria such as the German Medical Admission test “Test für Medizinische Studiengänge” (TMS) within their so-called individual quotas (Auswahlverfahren der Hochschulen, AdH). Each medical school in Germany can adjust its individual quota within certain parameters to fit their specific curriculum, thus providing them with some leverage to prioritize selection criteria, and it is used for allocation of 60% of German study places. At the time our data was collected, the other 40% of available medical school places in Germany was distributed centrally based on high school grades and waiting list. If successful with their applications, applicants enter medical school directly after graduating from high school. If they are not successful at first attempt, many applicants decide to apply again, some choosing to start vocational training in a medical profession instead. Vocational training in most medical fields in Germany (e.g. as a nurse or paramedic) does not usually take place at the university level and thus has more relaxed academically oriented entry-level requirements (e.g. GPA grades). Moreover, within the German medical admission system, professional experience is uniquely and narrowly defined as completed vocational training in a relevant medical field (e.g., nurse, paramedic) which renders it a comparable and thus generalizable criterion between different medical schools.

Other countries also consider prior professional experience, but rarely in initial selection stages and mostly rather vaguely defined as some relevant experience which makes a comparative evaluation of this criterion difficult. A common selection process involves multiple stages, where applicants are pre-selected based on cognitive criteria, and a subgroup is then invited to take part in more individualized selection processes such as structured interviews in the later stages. Professional experience is often considered in interviews, where, for example, paid or unpaid clinical work experience (e.g., community service) is assigned high importance relative to other criteria according to a 2008 survey among 120 admission deans from the US and Canada (Monroe et al., 2013). Many UK medical schools expect their applicants to have completed some work experience involving direct patient contact, and encourage them to further elaborate on these experiences during interview stages (Medical Schools Council, 2021).

However, evidence for the predictive validity of this criterion in addition to GPA and other main admission criteria, especially based on more generalizable multicenter data, is currently missing, and its use as a direct or indirect criterion is only justified by its high ecological validity and acceptability (Stegt et al., 2018).

The effect of prior professional experience as a selection criterion is not well understood in general. So far, it has mostly been discussed as either a means to achieve widened participation, for example by increasing the chance of admission for applicants who despite not having been high achievers at school could still be expected to succeed at medical school based on their motivation and experience (Powis et al., 2007), or equally as a factor potentially inhibiting access to medical schools for applicants with more diverse backgrounds, as prior work experience is ill-defined and difficult to obtain in many countries and might favor applicants whose parents are doctors as well (Medical Schools Council, 2021).

Prior professional experience has also been discussed as a factor contributing to medical students’ career choices (Bunker & Shadbolt, 2009). Some evidence points to the effects of work experience on students‘ decision to practice as a doctor in more rural areas, making work experience a potential leverage point to address shortages of doctors in these areas (Kesternich et al., 2017).

However, empirical data on the role of professional experience prior to study for actual study outcomes is scarce, conflicting and generally not based on multicenter approaches: Investigations based on data from Heidelberg Medical school revealed rather weak negative associations with outcomes within the first two years of study (which in Germany are considered pre-clinical and are designed to lay the theoretical foundations), and no associations with outcomes within the subsequent, more clinically oriented years of study (Hampe et al., 2009).

On the other hand, the authors of a small pilot study from Germany, found some evidence that professional experience as a criterion might add incremental predictive validity to GPA grades: 79% of students selected via the AdH quota were rated “absolutely suitable” for their profession by their supervisors in their final year of study as opposed to 42% of students who were selected via GPA only (Kötter et al., 2020).

From an educational point of view, prior professional experience in a medical field could affect study performance in different ways: Prior experience might lead to higher performance because prior clinical experience could help students make more sense of the knowledge obtained during their studies in a practical way and thus integrate it more quickly and efficiently (McManus et al., 1998).

However, higher perceived stress levels are usually associated with worse outcomes (Chisholm-Burns et al., 2021). Regarding professionally experienced medical students, this might go both ways: Indirect contextual factors such as parenthood, family or work obligations could lead to a perception of higher stress levels. This is also reflected in the finding that higher age at entry is usually associated with worse academic outcomes (Puddey & Mercer, 2014).

On the other hand, this particular group of students could experience less stress *within* their study curriculum because they have already worked in a medical field. They might therefore feel less in need to adjust to the stressors associated with it, and they might also have more knowledge of what to expect or how to behave in a clinical setting. Additionally, they might have developed better coping and life skills contributing to an enhanced resilience when compared to more unexperienced students.

Professional experience is used as an additional selection criterion beyond GPA grades, and results in the Medical Admission test TMS by a majority of German medical schools. This is because the GPA is considered to be the selection criteria with the highest and most stable validity, and its ability to predict study outcomes is well documented based on international data as well as data from German medical schools more specifically (e.g., Gold & Souvignier 2005 Hinneberg 2003; Kadmon et al., 2014; Salvatori, 2001; Trapmann et al., 2007). Moreover, the TMS is used as an additional cognitive criterion in the admission process of almost all German medical schools with very few exceptions, because of the high demand on medical study places relative to their availability.

The TMS was developed in the 1970s and 80s as a paper-pencil psychometric aptitude test and was designed to assess a number of cognitive abilities relevant to the medical field. These abilities can be grouped under the three dimensions “reasoning”, “visual-spatial information-processing”, and “memory” (Trost et al., 1998). Its predictive validity for study outcomes in the pre-clinical as well as clinical phases has been demonstrated in numerous studies, although these studies include no student cohort data after 2013 or any multicenter data including complete cohorts (Hänsgen & Spicher, 2001; Hell et al., 2007; Schult et al., 2019; Stumpf & Nauels, 1990; Trost et al., 1998).

Evidence for the incremental validity of the TMS, albeit based on monocentre data only, was also demonstrated by Kadmon & Kadmon (2016) who conclude that TMS results increase the predictive power of GPA results on study success and continuity for both students with top and inferior school leaving grades, and thus differentiate between potentially successful and less successful students in both GPA categories.

Therefore, we investigated the effect of prior professional experience of medical students beyond their GPA results and performance in the well-established admission test TMS on study outcomes within the first two years of study at two German medical schools, Heidelberg and Göttingen.

We especially drew on data from two different locations, not only to obtain more generalizable results, but also because of differences in selection procedures and curricula between medical schools, which might result in differing observed relationships between prior professional experience and study performance. At Heidelberg Medical school, for example, TMS and GPA results are integrated in a compensatory selection procedure, where a mediocre GPA can be compensated by an excellent TMS result or vice versa in the ranking position of an applicant. In contrast, applicants to Göttingen Medical school could increase their likelihood of being invited to an individual interview (the latter not being part of the Heidelberg selection process at all) by reporting their TMS score regardless of the result. Therefore, in Göttingen, as compared to Heidelberg, the likelihood is higher for applicants to report mediocre test results.

In sum, we addressed the following research questions in our study:

- Is prior completed vocational training in a relevant profession related to medical students’ study outcomes within the first two years of their studies (predictive validity)?
- More specifically, can any effects of prior completed vocational training in a relevant profession be observed beyond the predictive effects of GPA and TMS (incremental validity)?
- Are any effects of prior vocational training on study outcomes comparable between the two study locations Göttingen and Heidelberg (generalizability)?

## Methods

**Sample.** Retrospective admission and outcome data of N = 2969 medical students currently enrolled at either Göttingen (n = 640) or Heidelberg (n = 2329) Medical Schools beginning with the 2013 cohort and including the 2019 cohort was gathered. During this period, 40% of all available medical school places in Germany were centrally allocated based on high school grades and waiting time after maturation, whereas 60% of the places were allocated according to the AdH. Although all applicants provide information on whether they have completed vocational training or not, the criterion is only considered as part of the AdH. Only cases with complete data on all three selection criteria GPA, TMS and prior vocational training (yes/no) as well as on study outcomes up to and including the end of the second year of study were included in our analyses (N = 1063; n_Göttingen_=230; n_Heidelberg_=833).

At Göttingen Medical school, information about all three selection criteria were only available for the AdH. At Heidelberg Medical school, all selection quotas were considered (N = 2329; n_GPA_only_=295; n_AdH_=1378; n_waiting_list_=512; n_international_students_=107; n_other_=37), however, most students in the final subsample from Heidelberg had also been admitted via the AdH (N = 833; n_GPA_only_=64; n_AdH_=745; n_waiting_list_=11; n_international_students_=1; n_other_=12).

The project was undertaken as part of the research cooperation network “stav” (Studierendenauswahlverbund = “student selection network”, https://projekt-stav.de/) funded by the German Federal Ministry of Education and Research. Locations were included based on the availability of data to form a convenience sample.

The study was approved by the Institutional Review Boards of the Medical Faculties of the Universities of Heidelberg (application number S-027/2020) and Göttingen (22/11/18), respectively. It thus complies with the ethical standards established in the Declaration of Helsinki.

**Selection criteria.** GPA grades in our sample are expressed on the scale of the German baccalaureate with 1.0 as highest (best performance) and 4.0 as lowest possible grade. TMS results are indicated as percentile rank, i.e., percentage of test takers with equal or lower results. On average, students in our final sample had a GPA grade of 1.35 (SD = 0.54, Min. = 1.0, Max. = 3.7) and a TMS percentile rank of 89.94 (SD = 14.23, Min = 2.87 Max = 100).

Information on any completed vocational training in a relevant medical field was included as a binary code with 0 = *relevant vocational training not completed* and 1 = *relevant vocational training completed* (The list of relevant professions is almost identical between Heidelberg and Göttingen and includes the following: emergency paramedic; midwife; (pediatric, geriatric and srub) nurse; medical (lab and radiology) assistant, and assistant for functional diagnostics; physical, occupational and speech therapist. In Heidelberg some additional occupations include: orthoptist, biological or chemical laboratory technician). In total, n = 74 of the students in our sample (6.4%) had completed a relevant vocational training prior to their studies.

**Study outcome parameter.** As a common outcome parameter of the two medical schools with different curricula and exams, we defined the mean percentile rank over all exams of the first two years of study. Only students, who successfully completed the first two study years including all exams were included in the study. If more than one result due to failed exam attempts of a student were available, we used the best result of the successful attempt. The mean percentile rank over all exams was then converted to fit a normal distribution by use of the blom method (cf. McManus et al., 2013; Meyer et al., 2019).

**Controls.** In order to control for effects of demographics, we also included age and sex in our analyses because comparable studies had found effects of these demographics on study outcomes (Haist et al., 2000; Puddey & Mercer, 2014; Veloski et al., 2000). No other demographic variables (such as ethnicity or socio-economic background) were available due to strict data privacy laws in Germany.

**Statistical Analyses**. By use of hierarchical linear regression analyses, we assessed the effect of the three selection criteria GPA, TMS and vocational training as predictors and controlled for the effects of age and sex on our pooled study outcome parameter. We used regression analyses to be able to assess the effect of individual predictors on study outcomes independent of the other predictors. For example, negative simple correlations between vocational training and study outcome based on prior research could be explained by confounding effects with GPA or TMS scores: because applicants with prior professional experience generally achieve poorer TMS results than those who just graduated from high school, whereas TMS results positively correlate with study outcome, it appears necessary to adjust for these confounds (cf. Zimmermann et al., 2017).

**Difference between medical schools.** In a first step, we examined whether study outcomes significantly differed between the two medical schools Heidelberg and Göttingen. In this case, a multi-level approach is warranted due to data dependency. We calculated an intercept-only model with *medical school* as grouping variable and study outcome as dependent variable (DV) without any predictors to assess the intraclass correlation coefficient (ICC). The ICC thus obtained depicts the proportion of variance of the total variance explained by our Level-2-parameter *(medical school*), and, thus, quantifies the extent to which the data is dependent due to systematic differences in the DV between the schools.

All analyses were performed with R Version 4.0.2 with a significance level of α = 0.05.

## Results

**Fig. 1:**
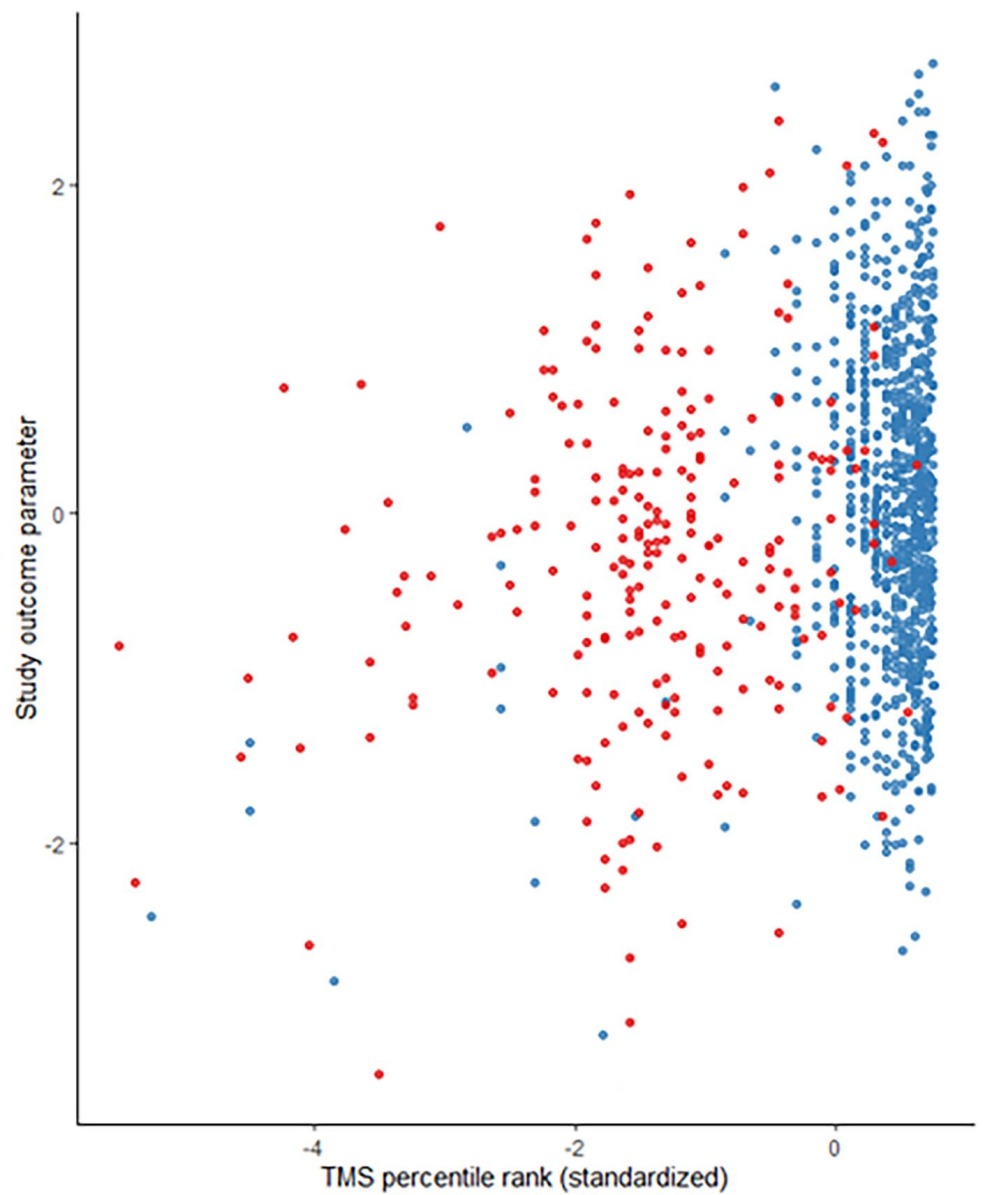
Study outcomes in relation to standardized TMS aptitude test percentile ranks, by medical school. Red dots = Göttingen, blue dots = Heidelberg

**Descriptives.** Table 1 shows an overview of the distribution of the predictors GPA, TMS, and vocational training, as well as age and sex, separately for Göttingen and Heidelberg, respectively. The subsamples are comparable with regard to age and GPA grades. Differences are apparent with regard to sex with a higher percentage of women studying in Göttingen, the TMS result, which is notably higher on average in the Heidelberg sample (M_Heidelberg_ = 93.3 vs. M_Göttingen_ = 67.6), and with regard to the proportion of students with vocational training: 19.1% of students in Göttingen vs. 3.6% of students in the Heidelberg sample. A graphical analysis additionally reveals the distributional differences of the TMS percentile ranks between the two medical schools (Fig. 1), indicating a notably higher variance in the sample from Göttingen.

**Table 1:** Distribution of students‘ socio-demographic characteristics and selection criteria

Variables	Heidelberg (*N* = 833)		Göttingen (*N* = 230)	
	Mean	SD	Mean	SD
Age	19.7	1.76	20.4	1.43
GPA (German “Abitur“)	1.33	0.34	1.45	0.21
TMS percentile rank	94.4	7.78	67.6	16.1
Vocational training (% yes)	3.6		19.1	
Sex (% female)	52.0		70.4	

Differences between medical schools were also found with regard to the study outcome parameter (Table 2), which showed lower performance scores in Göttingen compared to Heidelberg. Table 2 depicts standardized study outcomes separately by sex, vocational training (yes/no) and medical school. Comparing the absolute values without considering the effect of other factors, students with professional experience achieve poorer results than their fellow students without such experience. (M_vocational_training_=-0.233; M_no_vocational_training_=0.137). Accordingly, Table 3, which shows the correlation matrix with study outcomes and predictors, reveals a negative simple (bi-variate) correlation.

**Table 2:** Standardized study outcome means by sex, vocational training (yes/no) and medical school

Variables	Sex				Vocational training				Medical school			
	Female (n = 595)		Male (n = 468)		Yes (n = 74)		No (n = 989)		Göttingen (n = 230)		Heidelberg (n = 833)	
	Mean	SD	Mean	SD	Mean	SD	Mean	SD	Mean	SD	Mean	SD
Study outcome parameter	0.043	0.921	0.198	0.933	-0.233	1.01	0.137	0.918	-0.112	0.972	0.173	0.908

**Table 3:** Bi-variate pearson correlations between all predictors and study outcome parameter

Variable	2	3	4	5	6
1. GPA	-0.069*	0.351***	0.456***	-0.161***	-0.331***
2. TMS		-0.308***	-0.261***	-0.218***	0.199***
3. VT			0.401***	0.019	-0.101***
4. Age				-	-0.182***
5. Sex^a^					-0.083**
6. SOP					-

*Note.* GPA = German “Abitur” (unstandardized, 0 = best result), TMS = TMS percentile rank, VT = completed relevant vocational training (0 = no, 1 = yes), ^a^ = male (1), female (2, SOP = study outcome parameter (higher value = higher performance), * *p* < .05, ** *p* < .01 *** *p* < .001

**Multicollinearity**. Because individual predictors were correlated, which could hamper adequate interpretation of results, we checked for this important pecondition for regression analyses by calculating the variance inflation factor (VIF). With a VIF < 3 for all predictors, we assumed no problem with multicollinearity and therefore good interpretability of results.

**Effect of medical school.** With an ICC = 0.04430576/(0.04430576 + 0.98496147) = 0.043, 4.3% of the total variance in study outcomes is due to differences between locations. In a next step, we assessed whether this level-2-variance significantly differs from 0. We performed a likelihood-ratio model comparison between the intercept-only model and a simple linear model without the random effect of *medical school*. The difference was significant with χ² (*1*) = 13.246, p < .001. Therefore, we decided to use a multi-level approach for all subsequent analyses.

**Prediction of study outcomes.** In a multi-level approach with *study outcome* as DV and *medical school* as level-2-predictor we subsequently added each individual level-1-predictor (GPA, TMS, vocational training, age, and sex) as fixed effects only to see if any one addition significantly improved model prediction. We also tested for a possible interaction between age and vocational training. To this aim, we compared model fits between each model and its counterpart of higher model complexity to which one additional predictor had been added. This also means that in a first step, we allowed the level-1-predictors’ means (intercepts) to vary by medical school only.

An overview of model comparisons is given in Table 4.

**Table 4:** Effect on study outcome: Model comparisons

Mo-del	predictor	ß	*SE*	95% CI			*p*	AIC	BIC	χ² *(*∆ *df)*	∆ *p*
1	-	-	*-*	-		*-*		3011.2	3026.2		
2	GPA	0.321	0.029	0.263	0.378	***		2898.4	2918.3		
**Model 1 vs. Model 2**										**114.842(** ***1*** **)**	***
3	TMS	0.251	0.041	0.172	0.331	***		2863.4	2888.2		
**Model 2 vs. Model 3**										**37.049(** ***1*** **)**	***
4	VT	0.325	0.125	0.081	0.570	**		2858.6	2888.4		
**Model 3 vs. Model 4**										**6.762(** ***1*** **)**	**
5	Age	0.001	0.034	-0.066	0.068	0.977		2860.6	2895.4		
**Model 4 vs. Model 5**										**0.000(** ***1)***	**0.985**
6	Sex	-0.214	0.059	-0.330	-0.098	***		2847.6	2882.4		
**Model 4 vs. Model 6**										**12.998** ***(1)***	***
7	VT*Age	0.090	0.089	-0.084	0.264	0.310		2877.2	2921.8		
**Model 6 vs. Model 7**										**0.855** ***(2)***	**0.652**

*Note.* Random-Intercept Models with predictors as fixed effects, DV = study outcome parameter; N = 1063; GPA = German “Abitur”; VT = completed relevant vocational training; Model 1 = *Medical school* as varying intercept; Model 2 = Model 1 + *GPA* as fixed effect; Model 3 = Model 2 + *TMS* as fixed effect; Model 4 = Model 3 + *VT* as fixed effect; Model 5 = Model 4 + *Age* as fixed effect; Model 6 = Model 4 + *Sex* as fixed effect; Model 7 = Model 6 + Age*VT interaction as fixed effect; AIC = Akaike’s Information Criterion; BIC = Schwarz’s Bayesian Criterion; ∆ *p* = Significance of model difference; * *p* < .05, ** *p* < .01 *** *p* < .001

All selection criteria contribute to model prediction, the TMS result and vocational training significantly improve prediction of study outcomes above and beyond GPA, and GPA and TMS, respectively. Regarding demographics only the addition of *sex* to the model improved model fit, neither *age* nor a potential interaction between *age* and *vocational training* significantly improved prediction beyond the selection criteria.

In a next step, we also successively allowed regression weights to vary by location for each of the selection criteria. We were especially interested in medical-school-specific differences in the relationship between *vocational training* and *study outcome*. However, further improvement of the model could not be obtained. The final model, therefore, is a multi-level model with *medical school* as Level-2-predictor and GPA, TMS, vocational training, and sex as Level-1-predictors (Model 6 in Table 4). An overview of this model is given in Table 5. All predictors in the final model explain 14.5% of the total variance of the students’ study outcomes.

**Table 5:** Effect on study outcome: Final model with varying intercepts by *medical school*

Predictor	ß	*SE*	95% CI		*p*
Constant	0.384	0.171	0.048	0.720	0.025*
GPA	0.376	0.031	0.316	0.437	< 0.001***
TMS	0.255	0.042	0.172	0.337	< 0.001***
Vocational training	0.331	0.124	0.088	0.574	0.008**
Sex _a_	-0.214	0.059	-0.330	-0.098	< 0.001***

*Note.* Random Intercept Model with DV = study outcome parameter; N = 1063; a = male (1), female (2); * *p* < .05, ** *p* < .01 *** *p* < .001

**Fig. 2:**
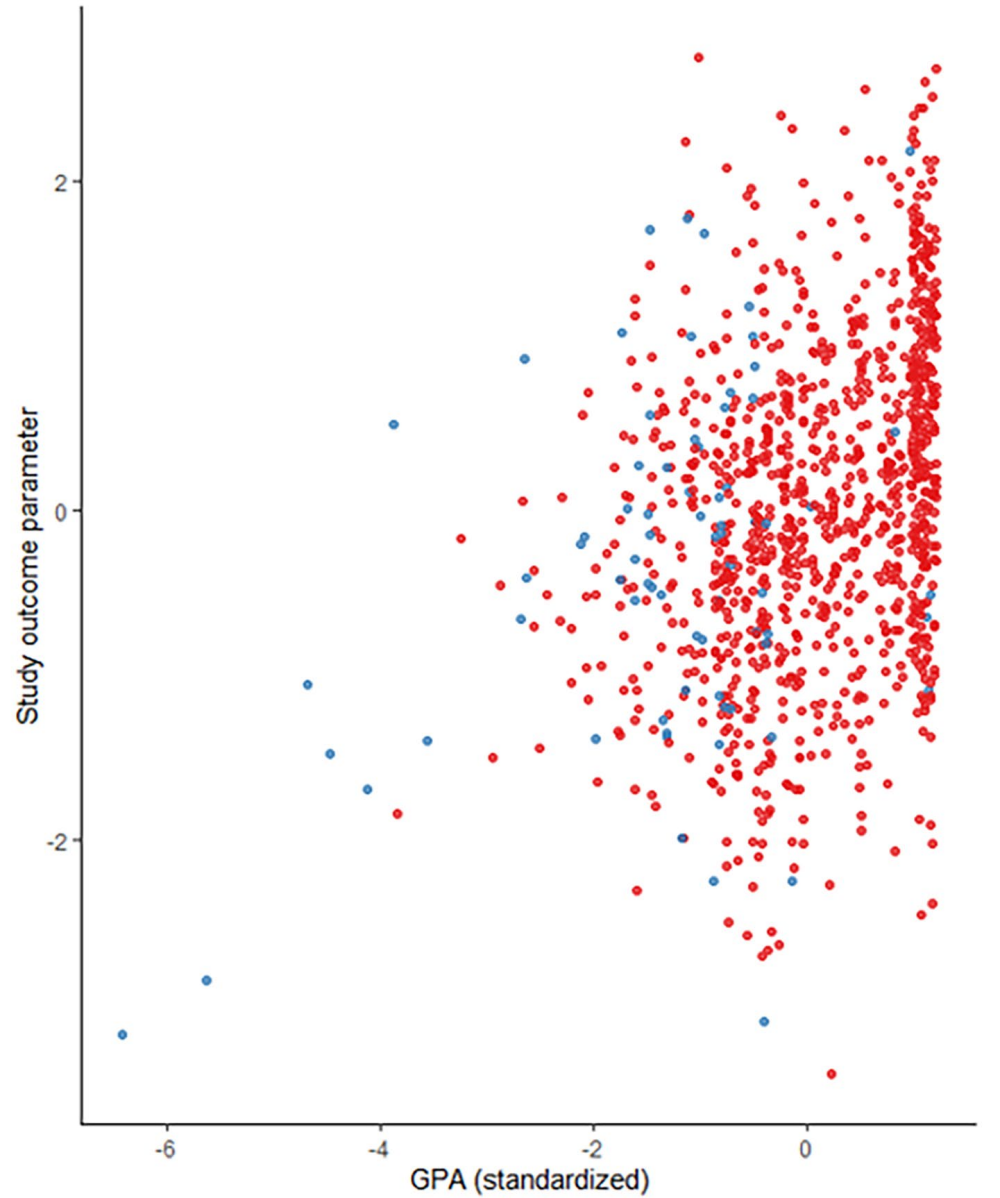
Study outcome as a function of GPA grades (standardized, German “Abitur”, 1 = best result) and completed vocational training (red dots=”no”, blue dots=”yes”)

A final graphical analysis (Fig. 2) revealed that among the students in our sample who had completed relevant vocational training prior to their studies, there were a few with comparably low GPA grades, and poorer study outcomes. However, professionally experienced students appear to perform at least as well as their inexperienced fellow students if they had comparably good GPA results alongside their experience.

## Discussion

Based on retrospective analyses of the study cohorts from 2013 to 2019, we were not only able to replicate the predictive validity of GPA grades and aptitude test results (TMS) on study outcomes of medical students at the two German medical schools Heidelberg and Göttingen. We were also able to provide first evidence that completed vocational training in a relevant medical field prior to medical studies predicts (cognitive) study outcomes pooled over the first two years of study, beyond the established selection criteria.

This finding supports an interpretation according to which professionally experienced medical students have gained important skills during vocational training which are conducive to succeeding at medical school and appear to also be independent of general life experience because we controlled for the factor age. These skills may include but may not be limited to stress coping skills or familiarization with and knowledge specific to the medical context.

Importantly, in Germany relevant work experience is narrowly defined as completed vocational training equalling roughly 3 years of professional experience including successfully completed qualifying examinations. In contrast to this, definitions in most other countries are rather vague where work experience could also mean 2 weeks of unpaid community service at a hospital. Based on our results, it seems justified to consider strengthening the role of professional experience as a selection criterion in other countries by clarifying its definition and formal use as a more direct criterion.

It might be argued that age-related maturity, rather than the specific skills and experiences obtained during vocational training is the decisive factor driving the positive effect of such training in our model. However, we believe this to be unlikely, since age independent of professional experience did not significantly predict study outcomes in our model. Also, a possible interaction with professional experience did not improve the model.

On the contrary: An additional argument against the “maturity hypothesis” can be derived from experiences of German medical faculties with the so-called “waiting time quota”. This quota allowed applicants to increase their chances to enter medical school solely based on the amount of time they had already been waiting for a study place. This quota is now gradually abolished as its predictive validity is highly questionable: Despite their increased age, students admitted via waiting time generally perform worse than their fellow students admitted through other quotas (Kadmon et al., 2014).

The common explanation for this previously observed performance gap is that during high school students obtain a necessary foundation of academic skills and knowledge to be successful at medical school. Waiting for sometimes up to several years for medical school entry then creates a gap which later needs to be caught up on. We therefore believe it is unlikely that age-related maturity can outweigh these skill-related disadvantages, and doubt it is the driving factor behind the positive effects of professional experience on study outcomes in our study.

Another possibility is that the skills and maturity obtained via *any* professional experience regardless of the *type* of experience is the driving factor for its positive effect on our study: based on our data, we cannot rule out this possibility, and future work would need to address this further. However, a similar argument might be put forth here that the disadvantages in academic skill created by a longer waiting time until university-level entry is achieved may be unlikely to be outweighed by any professional training. Most importantly, in Germany, vocational training in the medical field for the most part is much less academized than in other countries.

It is conceivable that the association of professional experience with study outcomes may even be more pronounced if not only (pre-clinical) cognitive exams are considered but also if the more clinical-practical study performance is considered – our findings therefore will likely become even more important given that in Germany, clinical-practical content will be increasingly incorporated into earlier stages of study with new legislative foundations for this already in place. At an international level, curricula are likewise often much more integrated with clinical-practical teaching modules early on.

Our results stand in contrast with the (albeit limited) prior research which showed no association with or even negative predictive validity for study performance (Hampe et al., 2009). This may in part be due to small sample sizes in earlier studies as depending on the medical school and its selection process, sample sizes of students with completed vocational training prior to studies are very small which hinders adequate analysis. This was also shown in our study where 3.6% of students in the Heidelberg sample had completed vocational training compared to almost 20% of students in the Göttingen sample, and thus also demonstrates the importance of a multicenter approach to tackle these kinds of questions.

Moreover, confounding associations with additional selection criteria such as GPA may mask any positive effects of professional experience: GPA grades appear to exert a bottleneck effect on study performance. Our results demonstrate that good GPA grades as proxies for cognitive ability appear to be a prerequisite for the ability to succeed in the predominantly cognitively oriented first two years of study. With comparably good GPA grades, students with vocational training completed prior to their studies do not appear to be disadvantaged in their studies compared to their fellow students who enter medical school directly after graduation from high school. On the contrary, according to our analyses, with comparably good GPA grades, students with vocational training perform even better than their colleagues without such training.

This finding demonstrates that simple mean comparisons or correlational analyses do not appear to be sufficient in modeling the complex interplay between factors affecting study performance within the rather heterogenous group of students with prior professional experience. A similar argument was put forth by Zimmermann et al., (2017), who demonstrated the effect of complex interplays between factors in the selection process of medical studies on analyses of validity by reference to several compensatory selection criteria.

When we modelled for medical school-specific differences in the association between any of the predictors (e.g. vocational training) and study outcomes, model fit was not improved. Theoretically, such location-specific differences would be unlikely for the cognitive criteria (GPA, TMS) which have been consistently demonstrated to be positively associated with performance outcomes regardless of context.

However, with regard to the influence of vocational training on study performance, differences between medical schools are conceivable. Differences in curricula or in the characteristics of the student cohorts due to differences in the selection process could lead to differential effects of prior professional experience on study performance. Differences may become even more pronounced if international medical schools or German medical schools with reformed curricula are considered for comparison – due to their higher emphasis on an integration of clinical-practical modules early on.

Our study was limited to only two medical schools due to data availability. However, it supports the results of previous monocentric studies and partly offsets the difficulties of sample size that generally are encountered with the more differentiated questions around the validity of vocational training for medical student performance. At the same time, overfitting becomes more likely with increased model complexity and indeed, our attempt at including predictors as random effects confirmed that there was no additional variance, while at the same time they showed perfect correlation of regression weights with the intercept, both of which can be interpreted as indications of overfitting.

This means that our result does not exclude the possibility that the effect of vocational training on study outcomes could vary by medical school and that the additional model complexity can only be reliably modeled with additional medical schools in the sample. This further emphasizes the need to draw on multicenter data for research on the validity of selection criteria.

Similarly, we already observed considerable differences in the distributions of selection criteria with the two medical schools in our sample. For example, examinations in Heidelberg are specifically designed to train students for the state examinations and place higher requirements on students to this aim. It therefore is more difficult for students in Heidelberg compared to those in Göttingen to obtain the same grades. Consequently, in our sample, the students from Heidelberg achieved lower study outcome scores on average (when controlling for the other criteria) than the ones from Göttingen.

Not only can a multicenter approach in this context enhance generalizability of results, it can also partly offset some of the known challenges of medical admissions research such as range restriction issues in the criteria as students are further selected from an already highly selective and high-performing applicant pool, and the data of applicants outside of this highly selective group is lost for analysis because they never enter medical school.

In our case, for example, this was true for the TMS results. At both medical schools, applicants can choose whether they indicate their TMS result as part of their application. But only in Heidelberg, each applicant’s TMS result is directly integrated with GPA grades to yield a ranking position. A good TMS result can compensate for a suboptimal GPA result, while a weaker one can worsen the ranking position which is why weak TMS results are generally not reported. In contrast to that, the role of the TMS result in the selection process of Göttingen medical school is purely additive rather than compensatory which yields a higher variance in the TMS criterion, thus offsetting the range restriction in the overall sample.

In sum, we strongly encourage additional research on the role of vocational training based on multicenter analyses. More specifically, further research could elucidate whether prior vocational training not only affects performance in the more cognitively oriented pre-clinical stages of medical study but also the more practically-oriented clinical ones. Outcomes can then also include non-cognitive and more complex parameters such as supervisor feedback or assessment of clinical skills in entrusted professional activities (EPAs, Ten Cate 2005) or OSCEs (Harden et al., 2015).

Lastly, we would like to emphasize that we do not wish to conclude from our study that every medical school applicant should necessarily have professional experience – rather, if they do, and cognitive ability is comparable to those without such training, they can absolutely be successful in their medical studies. Therefore, using professional experience as an additional criterion (where additional criteria are necessary because of the high applicant/study place - ratio), it may be a valid criterion under certain circumstances.

To further clarify the circumstances under which professional experience can be a valid and useful criterion, further research is warranted: Because students with prior professional experience form a more diverse population in terms of their life circumstances as well as study performance, an important research aim would be to identify any variables mediating the potential for challenges or failure within this group (e.g., motivation, curriculum-specific particularities, professional activities alongside their studies, or family planning).

## Conclusions

Based on our results, the use of completed vocational training in a relevant medical field as a selection criterion for medical school in addition to cognitive criteria appears to be warranted: students with such training perform better than their fellow students without such training if they have comparably good GPA grades. Further analyses, ideally based on multicenter data, should identify any additional factors leading to study success in the heterogenous group of professionally experienced students. Moreover, it remains to be seen whether prior professional experience can also positively predict relevant social-practical and communication skills during medical studies and later in professional life.
